# The Case of the Egyptian Frigate at Liverpool, with Remarks on the Causation of Fevers, &c.

**Published:** 1861-10

**Authors:** Gavin Milroy


### Art. II.—THE CASE OF THE EGYPTIAN FRIGATE AT LIVERPOOL, WITH REMARKS ON THE CAUSATION OF FEVERS, &c.

By Gavin Milroy, M.D., F.R.C.P.

The history of this vessel, and the events connected with her arrival in the early part of the present year, were altogether too important, in a public as well as in a professional point of view, for them to be allowed to pass out of memory with their occurrence; they well deserve a permanent place in medical literature for future reference and instruction. Fortunately, a detailed narrative of all the leading particulars of what took place at Liverpool was read at the last meeting of the Epidemiological Society, and will, it may be hoped, be published in their Transactions: it was from the pen of Dr. Duncan, the able Medical Officer of Health of that town.

As several of the most interesting questions of sanitary science, and of sanitary practice too, are involved in the circumstances of the case, it may be useful to devote a few pages to their consideration, in the hope of attracting more and more the attention of the profession to the great subject of preventive medicine. And, first, for a brief notice of the history of the vessel.

She arrived in the Mersey, in charge of a pilot, on the 22nd of February, with "a clean bill of health" from Alexandria, which she had left some weeks before. On being boarded by an ordinary Custom-house officer, this official was told by the captain that one man had died on the voyage, and that a few of the men were on the sick list with bowel complaints. The vessel was soon after moved into one of the docks. It was only then discovered that she was in a state of the most disgusting filth and nastiness; the stench in the between-decks was intolerable, and more than eighty of her crew (the entire number being about 300) were ill, many in a hopeless condition, with fever, dysentery, and frost-bites. The native doctor on board was among the number. There was every reason, too, to believe that several deaths had occurred during the voyage from the Mediterranean. The pilot admitted that he had never been well since he had gone on board, and felt certain that he should yet suffer for it. His fears were soon to be realized. Within a few days after the admission of the vessel into the dock, six deaths took place on board. At the same time, thirty-two of the worst cases were sent to the Southern Hospital; the poor creatures were indescribably filthy, and swarming with vermin. Most of them were suffering from the worst form of alvine Flux, attended at times with extreme prostration, hot skin, low muttering delirium, and involuntary discharges; some were affected with typhoid pneumonia, and others were frost-bitten in their lower extremities, gangrene of the toes and feet having taken place in three or four instances. That the poison of genuine Typhus existed among the sick cannot be doubted, although its presence in a distinct and uncomplicated febrile form was not recognised, in consequence of it being associated with the prevailing dysenteric affection, and masked in its outward manifestations by the swarthy complexion and begrimed skins of the Egyptians, from whom, moreover, no intelligible account of their sickness was obtainable. It would, indeed, have been strange if the febrific virus had not been there, for all the ordinary elements of its generation were in full force among the wretched crew, huddled together in the filthy, unaired between-decks of a foul ship, half-starved and worse than half-clothed. Fever, nascent or developed, simple or complicated, will, it may be taken for certain, be as infallibly developed in such an atmosphere as mildew will form on the damp walls of a shut-up room; nor will aught securely prevent its manifestation in some form or another among the victims of such exposure, but their prompt removal into a pure atmosphere, with a continuous, free, and ample aeration. Unless this last-named precaution be taken, the disease may spread, even after the removal of the sick from the primary focus, to other persons—more particularly to strangers—who are brought into close proximity with them.

But to proceed. Although the wards where the sick were placed seem to have been overcrowded, and the ventilation appears to have been decidedly imperfect, the change from the foul ship to the clean hospital was at once beneficial; most of the patients rapidly recovered; three only of the worst cases of dysentery proved fatal, one within a few hours after admission. But the hospital attendants did not escape the danger of breathing the infected atmosphere. Three of the medical officers, two nurses, and a porter of the establishment sickened with fever (typhus) within a week or so, I believe, of the admission of the Egyptians; two of the cases had a fatal result. Some of the patients, too, in other wards were attacked; one was a man who had been admitted several weeks before with fractured thigh. He recovered; but the chaplain, who had visited him and other patients, died after an illness of twelve days. And such was the alarm and anxiety caused by these events, that it was judged advisable to close the hospital for several weeks in order to have it thoroughly purified.

But the poisonous effects of the pestiferous ship were not confined to the hospital; and this part of the story is not less instructive than that just recorded. When the sick had been removed out of the vessel to the hospital, the rest of the crew—at least, all of them that had strength to walk—were sent to one of the public baths in the town to wash themselves, and get rid of as much of the filth and fœtor clinging to their bodies and clothes as possible. Between the 26th of February and the 1st of March, more than two hundred went to the baths. On the 2nd of March, one of the bath attendants was attacked with the first symptoms of fever; he died after twelve days' illness. In the following week, two other attendants sickened; they recovered. The baths, too, as well as the hospital, were closed for a time.

I must not omit to mention, that the pilot also had been taken ill at his own house, a few days after landing from the ship, with a bowel complaint followed by fever, of which he sank after between two and three weeks' continuance. He had said from the first, "We shall hear more of this ship yet!"

Besides the pilot, two other persons who had gone on board were attacked; but both recovered. Neither in their case, nor in that of any of the other persons attacked with the fever, whether in the hospital or at their own homes, did it spread to any member of their families or attendants.

Here ends the medical history of the ship as regards Liverpool; but the record of sickness and death connected with and arising from her, does not stop here. The crew, with the exception of eleven or twelve who remained in hospital, were shipped on board another Egyptian war steamer at Birkenhead, which sailed for Alexandria on the 15th of March. The crowding was, of course, extreme, for the vessel had her own crew as well as that of the frigate which remained behind for repair.* During the voyage, a great deal of sickness prevailed on board, and numerous deaths —by one account thirteen, by another account twenty-seven— had taken place before she reached Malta. On arrival there, the captain, three engineers, and one or two other persons, all Englishmen, were labouring under typhus fever. Many of the Egyptian sailors also were on the sick list; but whether they were affected with the disease in its distinct and simple form, or associated and commingled with dysentery or other more obvious malady, it is not possible, from the want of accurate information, to say. All the sick were removed to the Central Hospital on shore, where several died, among others Captain Lawson and another Englishman. While the frigate lay at Malta, an engineer resident there

* It has been stated that none of the workmen subsequently employed in repairing the frigate at Birkenhead experienced any ill effects from being on board; but, of course, before the repairs were commenced, the foul ship must have undergone a thorough cleansing and disinfection.

was engaged for several hours on board repairing the engines; he sickened with fever soon after leaving her, and the disease proved fatal. She left on the 3rd of April, and reached Alexandria on the 7th. Numerous fresh cases of disease occurred during the voyage; upwards of thirty sick were sent on shore on the day of arrival, suffering either from fever, or from dysentery, rheumatism, &c. It appears that Said Pacha, the Viceroy, went on board immediately afterwards, turned out all the crew, replacing them with fresh hands, and that after the ship was thoroughly cleaned she put to sea. After this, nothing more, of course, was heard about her.

And what now are the lessons to be drawn from the foregoing narrative? First of all, it adds another well-marked instance to a host of similar ones on record of genuine Typhus fever, as well as of malignant Dysentery, becoming developed spontaneously, or, in other words, being generated *de novo* among persons previously healthy when they are crowded together in a foul unrenewed atmosphere, charged with the noisome exhalations from their own bodies,—and especially when the aggravating influences of unwholesome or defective food and of physical and moral depression are in action at the same time. It is necessary that this truth should be clearly and unhesitatingly recognised by the profession, as there still seems to be an idea—not, indeed, in general distinctly or definitely avowed, but only implied under such expressions as a "specific poison," "essentially contagious," &c.—in the minds of many medical men, that a disease which possesses the property of self-propagation from the sick to the well must always arise from, or be somehow or other connected with, an antecedent example of its existence.

But this supposition is quite untenable as respects the case now under review. There was no disease whatever among the men when they left Alexandria, nor, I believe, for some time after sailing from Malta. Typhus is not common in Egypt. There can be little doubt but that dysentery was the disorder which broke out at first among the crew; the foul air, bad food, and insufficient clothing would inevitably produce that. Then the state of the between-decks would necessarily become worse and worse; and as the weather grew colder, the poor wretches would huddle together more and more to keep themselves warm, the atmosphere in which they were immersed becoming of course every day more putrescently abominable. This is the true medium for the breeding of the typhus poison, and in which all maladies acquire its impress and character. Ordinary dysentery then becomes a typhous dysentery, ordinary pneumonia a typhous pneumonia, and ulcers and common frost-bites then run into hospital gangrene. Such was the case with the crew of the Egyptian frigate, and such, too, it may be added, was the case, on a wider and more terrible scale, with the allied armies in the Crimea during the disastrous winter of 1854-5. It was the self-engendered element of typhus, superadded to the self-engendered element of scurvy, which was the true cause of most of the dreadful mortality throughout the whole of that memorable campaign. The two services are continually illustrating and throwing light upon each other as regards the ætiology of disease, and nowhere certainly will the student of our profession find more interesting and conclusive evidence respecting the genesis and the aggravating causes of many zymotic distempers than in the records of nautical and military medicine. These records deserve to be better known than they generally are; and assuredly no one acquainted with their contents can for a moment have a doubt on the point now under notice. Its truth, indeed, is a recognised verity. "Air," says Sir Gilbert Blane, in his classical discourse on the health of the British navy, "contaminated by foul and stagnant exhalations, particularly those from the living human body, is the ascertained cause of typhus fever—known also by the name of jail, hospital, and ship fever—which has been a more grievous and general source of sickness and mortality in the navy than even the scurvy;" and he adds, "the dysentery, which stands next in order in point of fatality, is also generated and propagated by the want of cleanliness and ventilation."* Bad food and insufficient clothing have only to be added, and then we have all the most deadly agencies at work together for the destruction of human life. The recent autobiography of Sir James MacGregor—unhappily far too meagre in professional memoranda—will give the reader some idea of the waste of strength

* It has been a matter of discussion whether the fever and the dysentery thus engendered under the same circumstances are to be regarded as generically distinct diseases, or whether they are only different results of the same morbific virus acting on diverse constitutions of body, and influenced it may be also by ethnological and climatic peculiarities. There seems to be often a sort of mutual balancing in the prevalence of fevers and fluxes. When and where the one are most abundant, the others are less so; and *vice versâ*. In some tropical countries, fevers are the more dominant endemic disease, and dysentery is but the occasional and partial one; in other similar countries, dysentery is the endemic *par excellence*, and fevers play but a subordinate part. Again, the outbreak of some bad fevers is preceded by an unusual prevalence of bowel complaints, which become less frequent and severe as the febrile epidemic becomes more completely developed. This has been remarked in the case of yellow fever epidemics. It has often been observed, too, that diarrhœal and dysenteric patients have escaped attacks of a prevailing fever. On the other hand, the sudden cessation of an alvine flux is apt to be followed by fever symptoms, as in the case of Asiatic cholera. But without pursuing the subject, I will only add that Sir John Pringle, and other experienced army and navy physicians of last century, were of opinion that patients affected with malignant dysentery are apt to communicate typhus fever as well as the dysentery to other persons near them. A like remark may be made as to hospital gangrene.

and life among our troops at the end of last century and the beginning of the present one, by the continual occurrence of typhus fever in consequence of crowding the men together like cattle in confined, ill-aired barracks and hospitals. One of the greatest difficulties with which Wellington had to contend, during the early years of the Peninsular war, arose from the enormous proportion of the sick in his army, attributable mainly to this cause.

What is true as to the spontaneous generation of typhus under the sort of conditions mentioned, holds equally true, allowing for modifying circumstances, in respect not only of typhoid or enteric fever, and of all alvine fluxes, but also of the malignant or pestilential fevers which appear in other climates and regions of the earth in certain years or seasons. That the genuine Oriental plague is liable at times to be engendered under the very same circumstances of unwholesomeness and squalid wretchedness as typhus is engendered in this country, was shown beyond all contradiction by the development, three years ago, of the disease in the wretched hovels of the half-starved Arabs at Benghazi, on the African coast, after an absence, be it remembered, of any trace of the pestilence for twelve or fourteen years at the least, and when, moreover, no trace of it existed in any other part of the Levant. The case must be regarded as a demonstrative one, even if there was no other evidence of the sort on the subject, which, however, is far from being so; and on this account it deserves a place in the repertory of ascertained and authentic medical facts. And so, too, with the pestilence of the warm regions of the New World. Every now and then Yellow fever is springing up in different spots and regions, independently of all traceable connexion with localities already infected,—not unfrequently, indeed, after considerable intervals of absence, but, nevertheless, almost invariably under the like local physical conditions to which reference has been made. Nautical and military experience on several occasions of recent years and in different places, as at Demerara, Brazil, Barbadoes, and more especially at Bermuda, appears to be quite decisive upon this point. That, for example, this fever became developed spontaneously in the crowded between-decks of the foul prison hulks at Bermuda in 1853, must be considered as an incontestable fact, as clearly and indubitably so, indeed, as the typhus on board the Egyptian frigate. And so it has been in a multitude of other instances both in the navy and in the merchant service. The fever in the *Eclair* appeared at first as the ordinary endemic remittent fever of the African coast, and subsequently lapsed, under the pernicious influence of an impure atmosphere on board, into the worst form of the malignant pestilence.

The second lesson taught us by the case of the frigate is, that certain diseases which are generated on board a foul vessel are apt to become highly contagious. Never was a stronger proof afforded of this fact than by the events of the present case. The only sure test of a disease being communicable, be it ever remembered, is when it spreads to healthy persons in attendance on, or in close proximity to, the sick after the latter have been removed away from the locality where they were attacked, and where the disease may have been engendered. The pilot and the two other persons at Liverpool, who caught the fever after having gone on board the infected vessel, cannot properly be regarded as indubitable instances in point; for they were exposed to the febrific polluted atmosphere which was the *fons et origo* of all the mischief. And so it is often on shore on visiting the abodes of the poor where a case of fever has sprung up; the other inmates of the house or a casual visitor may become subsequently affected not necessarily from the patient himself, but from the operation of the very same cause which primarily produced it. At any rate, there must always be some room for doubt as to the real and efficient cause of the spreading of the disease under such circumstances. But all hesitation must cease when the sick have been removed to a distance, and when the attendants, who have never visited or been near the spot where they had been taken ill, become subsequently attacked. Now, this was strikingly the case in respect of the medical and other attendants in the Southern Hospital, and also at the public baths. The poison of the fever must have been conveyed in the bodies, and most probably also in the foul clothes of the Egyptians, and been thus transmitted directly to the persons who were brought into intermediate intercourse with them. The fact cannot be gainsaid or explained away; it is patent as the noonday.

That so many of the attendants in the hospital were attacked would have indicated an extraordinary virulence in the contagious property of the fever, if the wards had been well ventilated, and the sick been duly dispersed. But that the contagion was really not more virulent than usual is conclusively shown by the circumstance that the disease did not spread to any of the inmates or attendants of the infected who were treated at their homes; affording thus another illustration of the facility with which the extension of the poison may be arrested by simple means always at our command. And so it is with the other forms of malignant fever to which allusion has been made. In respect of the yellow fever, a disease which, from its recent disastrous effects in our ships of war as well as in our merchantmen, deserves especial attention at the present time, the effects of free and ample aeration in a wholesome spot in reducing to zero its contagious property are even more prompt and decided than in respect to typhus.

Hence it is that in a well-ventilated hospital, the spreading of this fever from patients received from infected ships is all but unknown. Not a single instance has occurred of recent years at the large Marine Hospital of New York, in upwards of a thousand cases of the fever treated in its wards. No evidence can therefore be more decisive. That, however, the utmost risk is incurred by going on board an infected vessel is equally true in regard of yellow fever as of typhus fever. It may be remembered that, in the case of the *Eclair*, the pilot who boarded her as well as two or three medical officers who had volunteered their services to treat the sick on board, were speedily smitten with the fever and died; but that no sooner were all the crew, sick and well, removed out of her and placed in roomy, airy hulks than all trace of the disease disappeared. The case of the *Icarus* the other day at Port Royal affords, on the one hand, another striking instance of the extreme danger of persons going on board a fever-smitten ship, and on the other hand, of the little risk, if any, of the disease spreading to the immediate attendants on the sick in a well-aired and spacious hospital.

It is high time, surely, that the endless controversies of medical men on the subject of the contagion of this and other like fevers should cease; for they continue to bring discredit on medical habits of reasoning, if not upon medical truthfulness and impartiality in dealing with facts. The division of doctors into "contagionists" and "non-contagionists" has long appeared to me to be about as rational as would be the division of geologists at the present time into the old rival sects of Neptunists and Vulcanists. And might not the conduct of those medical men who can see nothing but contagion as the cause of the spreading of a disease, and of other medical men who, because they have satisfied themselves that the disease is apt to appear under certain external conditions independently of any traceable previous case, refuse to admit the operation of contagion at all, be fitly paralleled by the conduct of geologists, who still persisted in maintaining that all the phenomena of the earth's surface were referrible to the exclusive action of water or of heat.

If the logic of facts is to be worth anything in the guidance of our judgment in such matters, it will be difficult to come to any other conclusion except what appears to be the very obvious one, viz., that certain diseases are both generable *de novo*, and are also propagable by communication of their poison, under certain conditions, from one person to another. If such be the truth, the real point of practical importance is to determine, on every outbreak of the disease, the extent and potency of each of these exciting causes in the development and extension of the malady. Until medical men come to take this view of the subject, there will be no end, it seems to me, to the constantly recurring discussions and disputes on questions whose solution is surely quite as obtainable by patient observation and careful, candid deduction as any other questions within the domain of Natural History. There are few or any of the class of zymotic febrile diseases which might not be shown, upon tolerably good evidence, to possess at times, and under certain insalubrious conditions, the property of communicability; and, therefore, if instead of continually propounding the question—"Is such a disease contagious?" we were in future to be more in the habit of asking ourselves such questions as these—"When and under what circumstances did the property manifest itself?" and "What part did it play in the diffusion of the disease? was it relatively great, or was it relatively inconsiderable as compared with other agencies?"—there is every reason to believe that the discovery of useful truth would be promoted, and the value of professional opinion would be more generally esteemed.

In the case of the Egyptian frigate, there can, I should think, be scarcely any difference of opinion—viz., 1. That the fever was generated or self-engendered on board, and that its diffusion among the crew was mainly caused by the poisonous atmosphere from the crowding of them together in filth and misery; and, 2. That its extension to the attendants in the hospital and baths on shore was due to the transmission of contagious miasmata from the sick and their clothing.

Lastly, some useful lessons in sanitary practice may be gathered from the case we have been considering. It suggests very forcibly the prime and paramount importance of thorough aeration as the only reliable means for preventing the spread of fever when it has once become developed on board a ship. The between-decks of most vessels is, perhaps, the very worst place in the world for the detention of fever patients; nor can this be wondered at when we remember that, besides the great difficulty of obtaining and maintaining a continuous renewal of fresh air, there are generally some unpleasant emanations from the hold and bottom of a ship which must tend to increase still more the vitiation of the atmosphere that is breathed. And certainly nowhere have fevers proved more terribly destructive than at sea, in emigrant and troop ships; and occasionally also in our ships of war. Neither will such disastrous consequences be avoided in the future until the great principle be more generally recognised, and more assiduously acted upon, that the only safety, upon the appearance of sickness, consists in enlarging the breathing space to all on board to the utmost, and at the same time rendering the ventilation more complete, *especially at night, and during the hours of sleep*. The *dispersion* of the well and unattacked is of even greater importance, in a preservative point of view, than the *segregation* of the sick, however right and necessary this precaution also always is. It would be most useful if fever patients were more frequently accommodated, under proper shelter, on the spar-deck, where they can always obtain a free and pure atmosphere, without which it is in vain to talk of medical treatment, as has been over and over again proved by experience in every climate. An excellent arrangement for this purpose has recently been adopted in some of the West India mail steamers, on the recommendation of Dr. Wiblin, the enlightened superintendent of quarantine at Southampton, in the event of the occurrence of yellow fever on board. Canvas huts, capable of holding a couple of low iron bedsteads, can be rigged at once on deck immediately in front of the foremast, where the sick can be watched and tended without difficulty. This plan might be adopted with benefit in vessels of war, more especially in steamers, while every available spare foot of space on the spar and upper decks should be at the same time utilized for the wider distribution of the rest or unattacked portion of the crew, whenever that destructive malady threatens to prevail.

The spreading of fever to so many persons in the Liverpool hospital affords in itself pretty positive proof that the ventilation of the wards in that institution stands in need of improvement. There might, indeed, have been insufficient space allowed to each patient; for a thousand or twelve hundred cubic feet is certainly not too ample space under such circumstances. But it is always to be remembered that one half this extent, provided there be the means for maintaining a constant and uninterrupted renewal of the air throughout the twenty-four hours, is far more safe and salutary than the full extent with defective aeration. The construction and arrangements of hospitals and infirmaries have been pressed on the attention of medical men and of the public generally, by Miss Nightingale, with admirable good sense and ability, and certainly not before the instruction was wanted, as every one acquainted with many of our provincial and metropolitan hospitals must admit. The most common defect is in the windows; they are either badly placed, or are insufficient in size, or do not open aright and with ease, or perhaps only open in one or two small frames, as in that worst kind of all windows for hospitals, schools, &c., those on the Gothic or old English style of architecture, with stone divisions and latticed glass, to the exclusion generally both of free light and air.

In conclusion, I would suggest that the case of the Egyptian frigate shows that the sanitary police and sanitary superintendence over the shipping in our great commercial ports is far from being so efficient as the interests of the community require.

Dr. Duncan, the able medical officer of the medical committee of the town of Liverpool, remarked on this point in his first report:—"The medical officer is of opinion that there is no ground whatever for the apprehension that the disease is likely to spread through the town in consequence of what has occurred. But it is a matter worthy of consideration whether some arrangement ought not to be adopted for giving immediate notice to the proper authorities of the existence of disease among the crews of ships arriving in the port, in order that steps may be taken to prevent its further extension." That some arrangement of the sort is needed, both for the reason mentioned and also for the purpose of guarding against exaggerated alarms both among ourselves and in foreign countries, must strike every one. Such an event as that of the Egyptian frigate might have led to a stringent and prolonged embargo upon our commerce and general intercourse in many places abroad, more especially as the dreaded name of "plague" was at first actually applied to the disease. On much slighter grounds, an interdict of twenty, thirty, or forty days has often been imposed on all shipping arriving from a port where such an occurrence had taken place. But apart from this consideration, it is clear that much distress and evil would have been avoided had the proper precautionary measures been carried out on board, under the direction of an experienced medical officer, after he had ascertained by personal inspection the state of the ship and the crew, immediately upon her arrival. Such a responsible officer would, besides other precautions, not have allowed the sick to have been landed with all their filth and filthy infected clothes on them: previous ablution and clean clothing would of course have been enjoined. Neither would he have permitted the rest of the crew to have carried all their abominations on shore, to get rid of them in the public baths of the town. Much preliminary cleansing and purification might easily have been done on board. Moreover, the ship herself would have been subjected to no end of sluicing and disinfecting before she was admitted into a dock where there were other vessels. Everything, in short, indicates, as it seems to me, the great want of the presence of a sanitary medical officer, whose duty it should be not only to board and inspect all vessels arriving with any sickness among the crew or passengers, and to enjoin the adoption of the hygienic measures required alike for the benefit of the sick and others on board, and for the welfare of the community on shore, but also to maintain a supervision over the condition as to health of the shipping at all times,—on arrival, during stay, and before departure—in our great commercial ports. That there is a large amount — much larger than is generally imagined—of ill-health and positive disease, ending in premature disablement if not in death, in our mercantile marine, is well known to all whose attention has been drawn to the subject; nor is it less notorious but much, very much, of it might be easily avoided by timely discovery and attention. A very great deal might be prevented altogether, for many of the causes of the evil are abundantly obvious; and of these none is more potent than the defective and bad accommodation of the men. Without enlarging upon this important subject, I will only now remark, that if the practice of berthing the crew in houses upon deck, as is done in all the best American ships, were generally adopted in our merchant vessels, not only would the comfort of the men be greatly increased, but their vigour of health and their effectiveness for hard work would be in no small degree promoted. And as the mere temporary loss of the service of only one or two of the hands out of a merchantman's crew often comes to be of serious moment in a long voyage or during boisterous weather, the matter is clearly one of no small moment in a national point of view.

---

